# Lack of cross-resistance between non-steroidal and steroidal aromatase inhibitors in breast cancer patients: the potential role of the adipokine leptin

**DOI:** 10.1007/s10549-021-06399-x

**Published:** 2021-09-23

**Authors:** Nazli Bahrami, Shakila Jabeen, Andliena Tahiri, Torill Sauer, Hilde Presterud Ødegård, Stephanie Beate Geisler, Berit Gravdehaug, Laurens Cornelus Reitsma, Knut Selsås, Vessela Kristensen, Jürgen Geisler

**Affiliations:** 1 Department of Oncology, Akershus University Hospital, Lørenskog, Norway; 2 Department of Breast and Endocrine Surgery, Akershus University Hospital, Lørenskog, Norway; 3 Department of Clinical Molecular Biology (EPIGEN), Akershus University Hospital, Lørenskog, Norway; 4 Department of Pathology, Akershus University Hospital, Lørenskog, Norway; 5 Department of Cancer Genetics, Institute for Cancer Research, Oslo University Hospital, University of Oslo, Oslo, Norway; 6 Institute of Clinical Medicine, University of Oslo, Oslo, Norway

**Keywords:** Breast cancer, Aromatase inhibitor, Letrozole, Exemestane, Adipokine, Leptin

## Abstract

**Purpose:**

The aromatase inactivator exemestane may cause clinical disease stabilization following progression on non-steroidal aromatase inhibitors like letrozole in patients with metastatic breast cancer, indicating that additional therapeutic effects, not necessarily related to estrogen-suppression, may be involved in this well-known “lack of cross-resistance”.

**Methods:**

Postmenopausal women with ER positive, HER-2 negative, locally advanced breast cancer were enrolled in the NEOLETEXE-trial and randomized to sequential treatment starting with either letrozole (2.5 mg o.d.) or exemestane (25 mg o.d.) followed by the alternative aromatase inhibitor. Serum levels of 54 cytokines, including 12 adipokines were assessed using Luminex xMAP technology (multiple ELISA).

**Results:**

Serum levels of leptin were significantly decreased during treatment with exemestane (*p* < 0.001), regardless whether exemestane was given as first or second neoadjuvant therapy. In contrast, letrozole caused a non-significant increase in serum leptin levels in vivo.

**Conclusions:**

Our findings suggest an additional and direct effect of exemestane on CYP-19 (aromatase) synthesis presumably due to effects on the CYP19 promoter use that is not present during therapy with the non-steroidal aromatase inhibitor letrozole. Our findings provide new insights into the influence of clinically important aromatase inhibitors on cytokine levels in vivo that contribute to the understanding of the clinically observed lack of cross-resistance between non-steroidal and steroidal aromatase inhibitors in breast cancer patients.

**Trial registration:**

Registered on March 23rd 2015 in the National trial database of Norway (Registration number: REK-SØ-84-2015).

**Supplementary Information:**

The online version contains supplementary material available at 10.1007/s10549-021-06399-x.

## Introduction

The third-generation aromatase inhibitors (AIs) (anastrozole, letrozole and exemestane) are widely used to treat estrogen receptor positive (ER +) breast cancer in postmenopausal patients in all stages of the disease [1–6].

Anastrozole and letrozole belong to the type-I class of non-steroidal aromatase inhibitors binding competitively to the P450 part of the aromatase enzyme. In contrast, the steroidal, type-II class aromatase inactivator exemestane binds irreversibly to the substrate binding pocket (active site) of the aromatase enzyme [7–9]. The fundamental biochemical differences between non-steroidal and steroidal aromatase inhibitors are from a clinical point of view of particular interest as a “lack of cross-resistance” has been documented in several clinical trials [10–16], providing the rationale for the use of exemestane following disease progression during treatment with a non-steroidal compound like letrozole [17]. However, the precise explanation for the observed lack of cross-resistance between steroidal and non-steroidal aromatase inhibitors is still unknown and it has been suggested that a detailed understanding of this clinical phenomenon may potentially provide a new strategy to treat hormone-sensitive breast cancer [18, 19]. To investigate the fundamental differences in the effects caused by non-steroidal AIs and steroidal AIs in vivo, we designed the NEOLETEXE-trial [20]. The present manuscript reports the results of a preplanned cytokine-substudy of the NEOLETEXE-trial.

Adipose inflammation is increasingly recognized as a crucial factor in breast cancer carcinogenesis and progression [21, 22]. Typical consequences are hyperinsulinemia, elevated insulin-like growth factor I (IGF-I) levels, adipokine imbalances including leptin elevation as well as increased estrogen levels [21]. The adipokine leptin seems to play a pivotal role through binding to specific membrane receptors and inducing different signaling pathways, including the JAK/STAT, MAPK, IRS_1_ and SOC_3_ pathways [23]. Leptin is also involved in the regulation of body weight and is an important mediator of obesity as it controls food intake and energy balance by signals to the hypothalamus [24–26]. An excess of body fat mass increases the breast cancer risk, especially in postmenopausal women where estrogen production by adipose tissue through its own aromatase activity stimulates tumor progression [27]. Leptin is secreted by normal and malignant breast tissue and has been shown to enhance the expression of aromatase via promoter II and I.3 using an AP-1 motif [27]. A significant association between plasma sex hormones and plasma leptin levels has been reported [28, 29] and previous studies have shown that postmenopausal women with breast cancer have higher concentrations of plasma leptin [30, 31]. In a previous publication we could also show that plasma leptin levels are tightly correlated to the basic whole body aromatization in postmenopausal women [31].

In the present study, we evaluated the serum levels of 54 cytokines, including all 12 known adipokines, relative to given treatment with letrozole and exemestane in a neoadjuvant setting, aiming for a direct head-to-head comparison in vivo. We hypothesized that the different effects of letrozole and exemestane on the expression of crucial adipokines like leptin may play a key role in the mentioned lack of cross-resistance between type-I and type-II aromatase inhibitors. Importantly, our study was not primarily designed to compare the clinical effects on the breast cancer tumors as both drugs have been shown to cause comparable tumor shrinkage in the neoadjuvant setting in previous trials.

## Materials and methods

### Trial design and patients

**Fig. 1:**
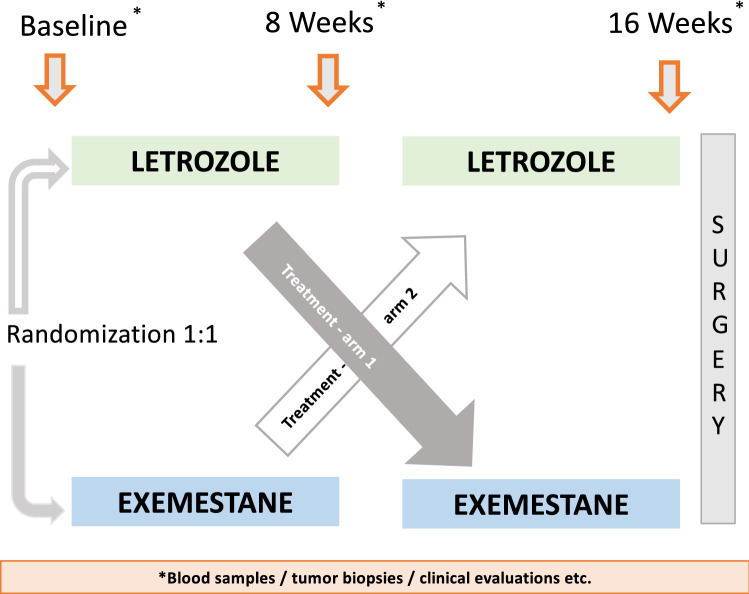
Study design—the NEOLETEXE study

The NEOLETEXE-study (Fig. 1) is a neoadjuvant, randomized, open-label, intra-patient, cross-over trial with the intention to study the effects of sequential aromatase inhibition by type-I aromatase inhibitor letrozole and type-II aromatase inactivator exemestane in vivo. The trial has been approved by the Regional Ethics Committee of South-East Norway (project number 2015/84) [20]. In this particular sub-study, a total of 39 postmenopausal women, all diagnosed with locally advanced, ER-positive and HER-2 negative primary cancer were included (Table 1). Locally advanced breast cancer (LABC) was defined as either T3-T4 and/or N2-3 primary breast cancer. However, patients with tumors above 4 cm but below 5 cm in diameter (“large T2-tumors”) were also includable in accordance with the international trend to provide neoadjuvant therapies to these patients in clinical trials. We defined postmenopausal status as age above 55 years or age above 50 years and at least 2 years of amenorrhea in addition to LH-, FSH- and plasma estradiol levels in the postmenopausal range.

**Table 1:** Patient characteristics

Patient	Age	BMI	cTNM	Type	ER (%)	PG	HER-2	Treatment
1	66	28	T4N1M0	NST	> 50	Negative	Negative	LET-EXE
2	70	22	T4N1M0	NST	> 50	Negative	Negative	EXE-LET
3	76	24	T3N1M1	NST	> 50	Negative	Negative	LET-EXE
4	64	28	T4N0M0	ILC	> 50	Negative	Negative	EXE-LET
5	61	27	T3N0M0	NST	100	> 10%	Negative	LET-EXE
6	81	26	T4N0M0	ILC	100	15%	Negative	EXE-LET
7	87	23	T4N0M0	NST	> 50	> 10%	Negative	LET-EXE
8	82	26	T4N1M0	NST	100	Negative	Negative	EXE-LET
9	73	28	T4N0M0	NST	90–100	> 90%	Negative	LET-EXE
10	80	27	T4N0M0	NST	> 50	> 10%	Negative	EXE-LET
11	87	28	T4N0M0	NST	> 50	> 90%	Negative	EXE-LET
12	78	31	T4N0M0	NST	> 50	Negative	Negative	LET-EXE
13	62	27	T4N0M0	NST	> 50	> 10%	Negative	EXE-LET
14	62	22	T4N0M0	ILC	> 50	> 10%	Negative	LET-EXE
15	84	29	T3N1M0	NST	> 50	> 10%	Negative	EXE-LET
16	83	30	T4N0M0	NST	100	> 50%	Negative	LET-EXE
17	62	25	T4N1M0	NST	> 50	> 10%	Negative	EXE-LET
18	85	23	T4N0M0	ILC	100	100%	Negative	LET-EXE
19	77	24	T4N0M0	NST	100	90%	Negative	EXE-LET
20	71	28	T4N0M0	NST	> 50	> 10%	Negative	LET-EXE
21	76	30	T3N1M0	ILC	> 50	Negative	Negative	EXE-LET
22	67	36	T4N0M0	NST	> 50	> 10%	Negative	LET-EXE
23	83	31	T4N0M0	IAC	> 50	> 10%	Negative	EXE-LET
24	89	24	T4N0M0	NST	> 50	> 10%	Negative	LET-EXE
25	82	25	T3N0M0	NST	> 50	> 10%	Negative	EXE-LET
26	83	35	T4N0M0	NST	> 50	> 10%	Negative	LET-EXE
27	70	28	T4N1M1	NST	100	> 50%	Negative	EXE-LET
28	71	30	T4N0M0	NST	100	> 50%	Negative	LET-EXE
29	73	31	T4N0M0	ILC	100	> 50%	Negative	EXE-LET
30	67	28	T3N0M0	SNEC	100	100%	Negative	EXE-LET
31	74	35	T3N0M0	ILC	100	100%	Negative	LET-EXE
32	78	25	T4N1M0	ILC	> 50	> 10%	Negative	EXE-LET
33	80	18	T4N1M0	SNEC	100	100%	Negative	LET-EXE
34	80	21	T4N0M0	ILC	100	100%	Negative	EXE-LET
35	78	27	T3N0M0	ILC	100	> 50%	Negative	LET-EXE
36	73	34	T4N0M0	NST	> 90	> 10%	Negative	EXE-LET
37	79	22	T4N0M0	ILC	> 50	Negative	Negative	LET-EXE
38	79	32	T4N1M0	ILC	100	20–30%	Negative	EXE-LET
39	70	32	T4N0M0	NST	> 50	> 10%	Negative	LET-EXE

*BMI* Body Mass Index, *cTNM* clinical and radiological examination of tumors size (T), lymph node status (N) and distant metastases (M), *ER* estrogen receptor, *EXE-LET* treatment sequence (neoadjuvant): exemestane followed by letrozole, *HER-2* human epidermal growth factor receptor 2, *ILC* invasive lobular carcinoma, *IAC* invasive apocrine carcinoma, *LET-EXE* treatment sequence: letrozole followed by exemestane (neoadjuvant), *NST* invasive carcinoma of no special type (historical term: invasive ductal carcinoma), *PGR* progesterone receptor, *SNEC* solid neuroendocrine carcinoma

Patients were randomized to neoadjuvant endocrine therapy (NET) by one of two treatment arms (Fig. 1).

#### Treatment arm 1

Letrozole (Femar®) 2.5 mg o.d. for at least 8 weeks, followed by exemestane 25 mg o.d. for another 8 weeks prior to surgery.

#### Treatment arm 2

Exemestane (Aromasin®) 25 mg o.d. for at least 8 weeks, followed by letrozole 2.5 mg o.d. for another 8 weeks prior to surgery.

Collection of blood samples, breast tumor biopsies, MRI evaluations and clinical examinations were performed at baseline (before initiation of any therapy), following at least 2 months on the first AI treatment (directly before switching to second treatment) and, finally, directly prior to surgery.

### Cytokine multiplex profiling

In total 54 cytokines including all 12 adipokines, IL10 family cytokines, IL6 and its receptors, IFN, tumor necrosis factor (TNF) superfamily cytokines, growth factors, osteokines and selected matrix metalloproteinases (MMPs) were analyzed in serum samples obtained from 39 postmenopausal breast cancer patients treated according to the NEOLETEXE-protocol. Blood samples were obtained at three timepoints: at baseline and following at least 2 months and 4 months on therapy with the two individual aromatase inhibitors given in a randomized sequence as monotherapy. The multiplex profiling platform Luminex 200 was used for quantitative analysis of the cytokines. We used three commercially available cytokine panels from Bio-Rad and one panel from Millipore. Panels from Bio-Rad included: 37-plex Pro Human Inflammation Panel 1 (lot# 64161726), Pro Human Adiponectin 1-plex Panel (lot #10010747) and 15-plex including 10-plex Pro Diabetes Panel (lot #64065098) plus five cytokines: IL6, IP10, VEGF, PDGFbeta and TNFalpha (standards lot# 64103329). The interleukin 24 (IL-24) single plex panel was purchased from Millipore (lot#HCYP4MMAG-64K-IL-24). Signal intensities of protein concentration for all cytokines were determined in a series of control standard dilutions to create standard curves as instructed by the manufacturers. The total serum protein level for each cytokine was assessed according to their locations on the standard curves using Bio-Plex Manager 6.0 (Bio-Rad). Data were exported to excel sheets for further analysis.

### Statistical analysis

All analyses were performed after natural log transformation of the observed data using IBM SPSS Statistics 25 and Excel 2016. Cytokine levels were analyzed in relation to categorical clinical parameters using ANOVA, Mann–Whitney-U (MWU), Kruskal–Wallis and *t*-tests. Unless otherwise stated, results were considered statistically significant, if the two-sided *p*-value was < 0.05. The cytokine correlation analysis was performed for pretreatment (baseline) log-transformed serum levels using Spearman correlations. Visualization of all finding was performed using either IBM SPSS Statistics 25’s graphic elements or by use of Excel 2016.

## Results

We performed a serum level variation analysis for 54 cytokines from samples collected from 39 postmenopausal patients with locally advanced, ER positive, HER-2 negative breast cancer. Cytokine serum levels were measured using multiplex array system Luminex xMap and observed values were log normalized.

**Fig. 2:**
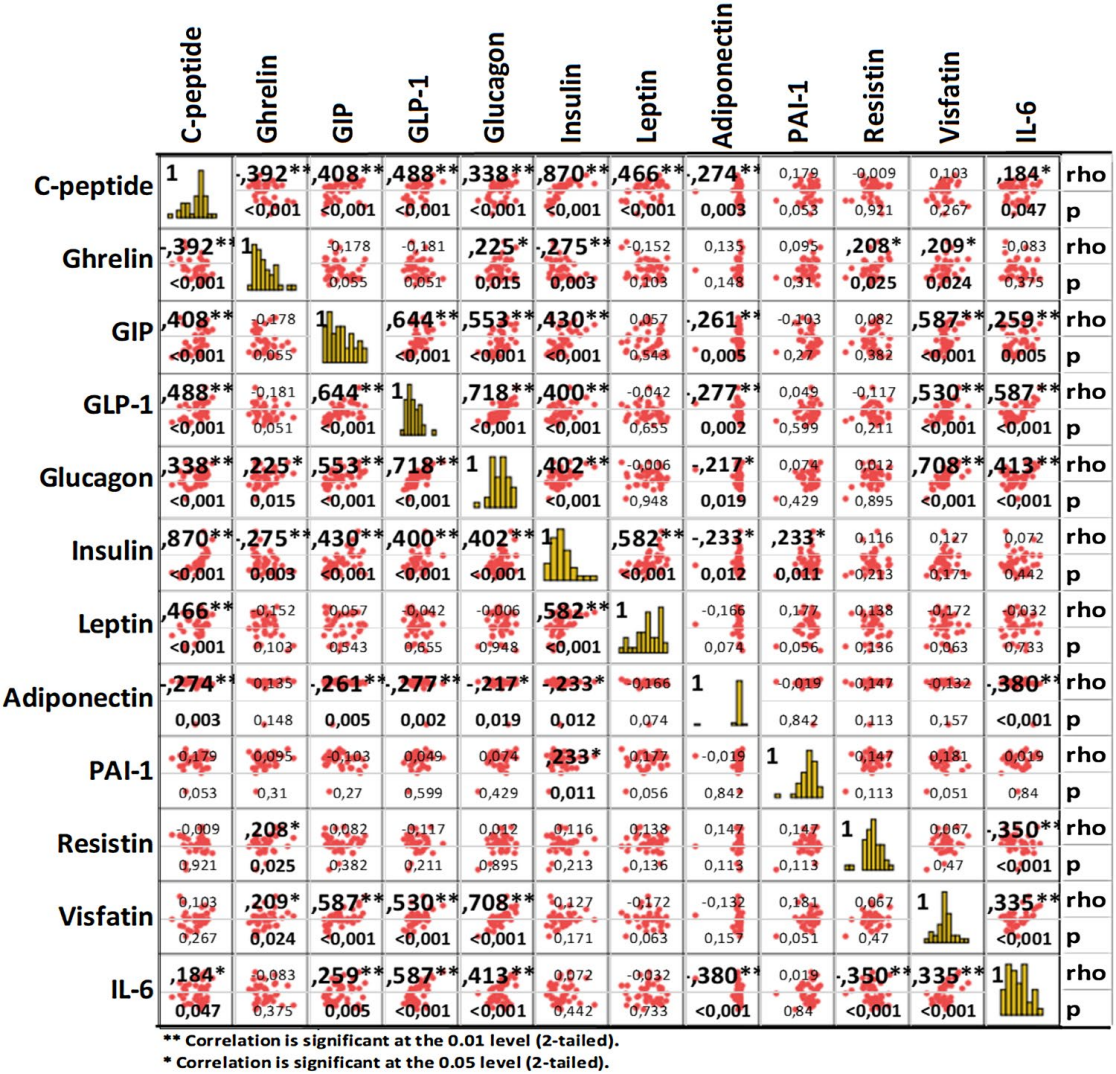
Spearman correlations between baseline serum levels of adipokines. Spearman correlations between serum levels of 12 adipokines (*n* = 39, timepoint: baseline) are shown by correlation dot plots, as well as by correlation coefficient values (rho) with significance (*p*) values. Significant *p* values are highlighted bold

The log-transformed cytokine levels for all patients and the influence of treatment with letrozole and exemestane, respectively (given as differences to baseline values) are summarized in Table 2. Statistical analyses were performed using normalized serum levels values of the cytokines against clinical parameters and observations. The overall results of our findings illustrated in groups or families of cytokines which are put together according to their common function, origin, or both are given in Table 2. Spearman correlations were calculated between all adipokine baseline values as summarized in Fig. 2. The correlations between adipokines are also given for the individual treatment arms over time in Supplementary Fig. 1.

**Table 2:** Variations in cytokine levels during therapy with letrozole or exemestane (differences relative to baseline levels)

Grouping	Cytokines	*N*	Mean (log) levels			Letrozole			Exemestane			One way ANOVA Drug category- (Post-drug—BL)* (*p*)	One way ANOVA category- variation Ltz vs. Exe (*p*)*
						Mean varied levels relative to BL	95% Confidence Interval for Mean		Mean varied levels relative to BL	95% Confidence Interval for Mean			
			Baseline (BL)	Letrozole	Exemestane		Lower Bound	Upper Bound		Lower Bound	Upper Bound		
Adipokines	C-peptide	39	7.11	7.05	7.06	− 0.06	− 0.18	0.06	− 0.05	− 0.13	0.03	0.923	0.477
	Ghrelin	39	5.72	5.76	5.77	0.03	− 0.04	0.11	0.05	− 0.04	0.13	0.839	0.671
	GIP	39	6.27	6.30	6.25	0.03	− 0.17	0.22	− 0.02	− 0.19	0.15	0.738	0.614
	GLP-1	39	5.42	5.46	5.48	0.05	− 0.03	0.12	0.07	− 0.01	0.14	0.745	0.195
	Glucagon	39	6.79	6.76	6.77	− 0.03	− 0.08	0.02	− 0.02	− 0.05	0.02	0.727	0.562
	Insulin	39	6.37	6.27	6.14	− 0.10	− 0.29	0.09	− 0.23	− 0.38	− 0.07	0.307	0.658
	Leptin	39	9.20	9.23	9.03	0.03	− 0.10	0.15	− 0.17	− 0.28	− 0.06	**0.019**	**0.000**
	PAI-1	39	10.84	10.84	10.83	0.00	− 0.13	0.13	− 0.01	− 0.14	0.12	0.878	0.411
	Resistin	39	9.07	9.12	9.07	0.05	− 0.06	0.17	0.00	− 0.07	0.08	0.471	0.028
	Visfatin	39	9.40	9.37	9.41	− 0.03	− 0.12	0.06	0.01	− 0.09	0.11	0.523	0.316
	Adiponectin	39	15.54	16.11	15.36	0.57	− 0.49	1.62	− 0.18	− 0.46	0.09	0.169	0.189
	IL-6	39	1.12	0.92	1.16	− 0.20	− 0.50	0.11	0.04	− 0.14	0.22	0.187	0.068
TNF family	TNF-a	39	2.87	2.85	2.94	− 0.03	− 0.10	0.05	0.06	− 0.02	0.15	0.109	**0.002**
	sTNF-R1	38	8.26	8.35	8.33	0.09	0.03	0.14	0.07	0.01	0.13	0.641	0.785
	sTNF-R2	38	7.02	7.15	7.15	0.13	0.06	0.20	0.14	0.07	0.21	0.918	0.304
	sCD30/TNFRSF8	38	5.92	5.99	6.17	0.07	0.01	0.13	0.25	0.15	0.35	**0.003**	**0.000**
	BAFF/TNFSF13B	38	9.11	9.16	9.20	0.05	0.00	0.10	0.09	0.03	0.16	0.259	**0.006**
	APRIL/TNFSF13	38	12.02	12.10	12.09	0.08	0.04	0.12	0.08	0.03	0.13	0.906	0.364
	LIGHT/TNFSF14	38	1.95	2.07	1.82	0.12	− 0.58	0.82	− 0.18	− 0.81	0.46	0.528	0.665
	TWEAK/ TNFSF12	38	5.90	5.95	5.91	0.05	0.01	0.08	0.01	− 0.04	0.06	0.251	0.223
IL-10 family	IL-10	38	2.49	2.45	2.61	− 0.04	− 0.37	0.29	0.12	− 0.02	0.26	0.360	0.988
	IL-11	38	0.54	0.74	0.56	0.20	0.00	0.40	0.02	− 0.30	0.34	0.337	**0.012**
	IL-19	38	− 2.46	− 2.11	− 2.33	0.35	− 0.76	1.46	0.14	− 1.11	1.38	0.795	0.646
	IL-20	38	0.80	0.69	0.37	− 0.11	− 0.36	0.14	− 0.42	− 0.98	0.14	0.304	0.469
	IL-22	38	− 3.22	− 3.41	− 3.27	− 0.19	− 0.91	0.53	− 0.05	− 0.69	0.58	0.769	0.477
	IL-26	38	6.01	6.06	6.13	0.05	− 0.03	0.13	0.13	0.05	0.20	0.152	0.316
	IL-27(p28)	38	2.61	3.13	2.95	0.52	0.14	0.90	0.33	− 0.59	1.26	0.705	0.195
	IL-28A/IFN-lambda 2	38	2.70	2.76	2.69	0.07	− 0.03	0.16	− 0.01	− 0.13	0.11	0.307	0.105
	IL-29/IFN-lambda 1	38	4.67	4.56	4.50	− 0.11	− 0.58	0.36	− 0.17	− 0.73	0.38	0.859	0.516
MMPs	MMP-1	38	6.45	6.55	6.40	0.10	− 0.02	0.22	− 0.05	− 0.22	0.11	0.139	**0.023**
	MMP-2	38	9.62	9.73	9.76	0.11	0.00	0.23	0.15	0.04	0.25	0.678	0.522
	MMP-3	38	7.72	7.67	7.85	− 0.04	− 0.18	0.10	0.14	− 0.02	0.29	0.084	**0.003**
Diverse cytokines	sCD163	38	11.48	11.54	11.49	0.05	0.00	0.10	0.00	− 0.06	0.07	0.245	0.393
	Chitinase 3-like 1	38	9.07	9.17	9.07	0.10	0.02	0.18	0.00	− 0.05	0.06	0.046	0.196
	gp130/sIL-6RBeta	38	10.46	10.48	10.46	0.02	− 0.03	0.07	− 0.01	− 0.06	0.05	0.451	0.080
	IFN-alpha2	38	2.99	3.15	3.05	0.16	− 0.01	0.33	0.06	− 0.56	0.68	0.752	0.067
	IFN-beta	38	3.23	3.37	3.11	0.14	0.03	0.26	− 0.12	− 0.55	0.31	0.237	**0.010**
	IFN-gamma	38	3.32	3.42	3.38	0.10	0.01	0.18	0.06	− 0.04	0.15	0.540	0.062
	IL-2	38	− 0.20	0.49	0.41	0.69	0.04	1.35	0.61	− 0.35	1.58	0.893	0.478
	sIL-6Ralpha	38	8.63	8.66	8.64	0.03	0.00	0.07	0.01	− 0.03	0.05	0.500	0.628
	IL-8	38	0.85	1.55	1.26	0.70	− 0.16	1.57	0.41	− 0.72	1.54	0.681	0.187
	IL-12(p40)	38	4.62	4.69	4.61	0.08	− 0.01	0.16	− 0.01	− 0.13	0.12	0.289	**0.023**
	IL-12(p70)	38	− 0.55	0.13	− 0.27	0.68	0.18	1.18	0.28	− 0.48	1.04	0.376	0.201
	IL-32	38	− 1.27	− 0.42	− 0.64	0.86	− 0.28	1.99	0.63	− 0.72	1.98	0.798	0.199
	IL-34	38	− 4.61	− 4.61	− 4.61	0.00	0.00	0.00	0.00	0.00	0.00	n.a	na
	IL-35	38	2.55	3.07	3.21	0.52	− 0.56	1.60	0.66	− 0.47	1.78	0.856	0.700
	IP-10	39	6.48	6.50	6.60	0.02	− 0.09	0.14	0.12	0.00	0.24	0.255	0.135
	PDGF-bb	39	8.06	8.00	7.98	− 0.06	− 0.14	0.02	− 0.08	− 0.21	0.06	0.865	0.956
	VEGF	39	2.14	2.71	2.76	0.56	− 0.21	1.33	0.62	− 0.07	1.30	0.910	**0.017**
	Osteocalcin	38	8.11	8.22	8.23	0.11	0.01	0.21	0.12	0.03	0.22	0.862	0.570
	Osteopontin (OPN)	38	10.10	10.26	10.33	0.17	0.08	0.26	0.24	0.15	0.33	0.271	0.118
	Pentraxin-3	38	6.71	6.74	6.73	0.03	− 0.09	0.14	0.01	− 0.09	0.12	0.864	0.159
	TSLP	38	3.20	3.34	3.31	0.14	0.01	0.27	0.11	− 0.02	0.24	0.723	0.213
	IL-24	39	− 4.41	− 4.37	− 4.43	0.04	− 0.23	0.31	− 0.03	− 0.22	0.16	0.679	0.467

*ANOVA for cytokine level variations post each drug relative to baseline, regardless of timepoint and treatment arm; two-sided *p*-value < 0.05 was considered as statistically significant

**ANOVA for cytokine level variations relative to start of drug administration [(levels after 8 weeks minus baseline levels) and (levels at 16 weeks minus 8 weeks levels)]-post letrozole vs post exemestane variations

**Fig. 3:**
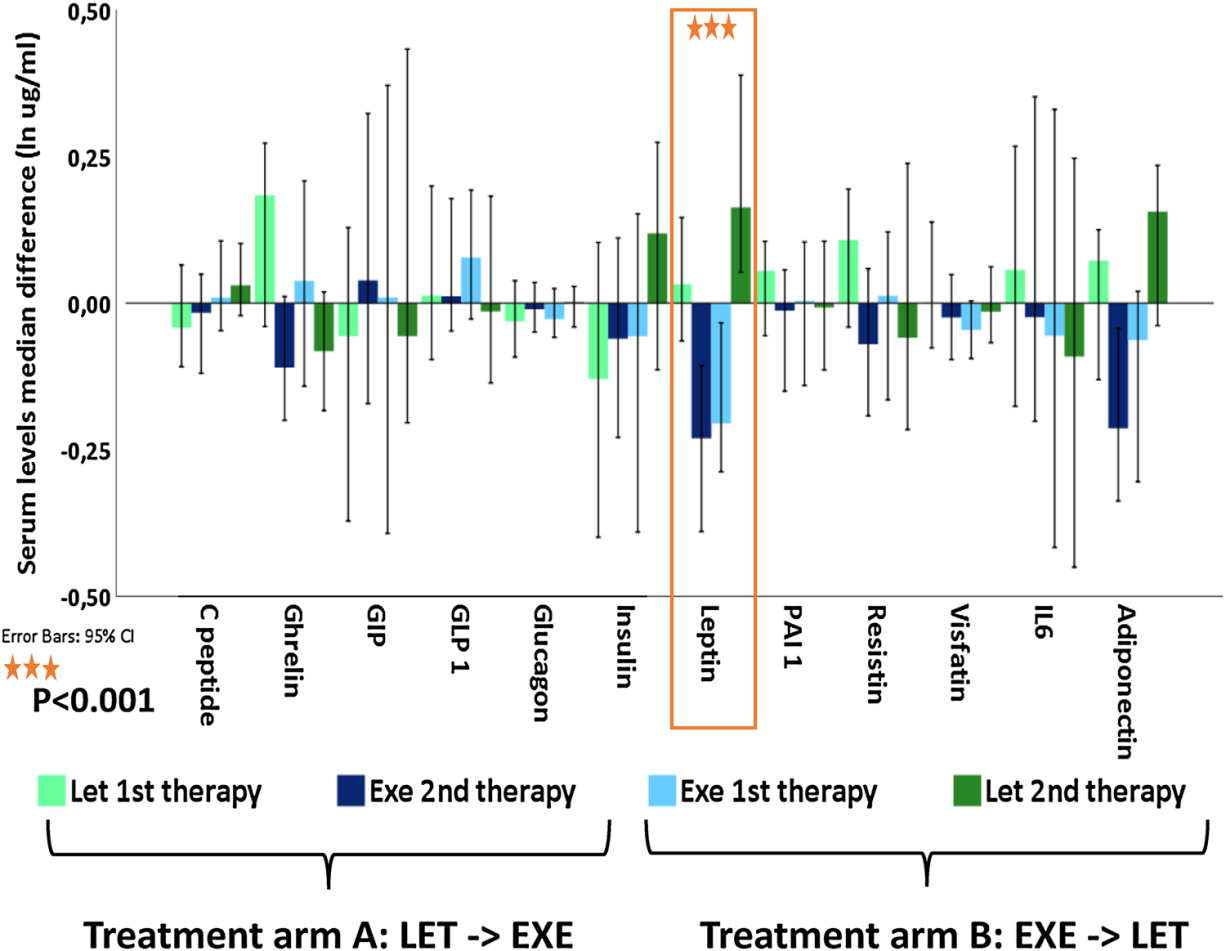
Influence of treatment with letrozole and exemestane on serum adipokine levels. Bar plot representing median log serum levels difference (y-axis) of 12 adipokines (x-axis) in breast cancer patients (*n* = 39), relative to neoadjuvant drug type and therapy time-point (color categories). Error bars represent 95% confidence interval

**Fig. 4:**
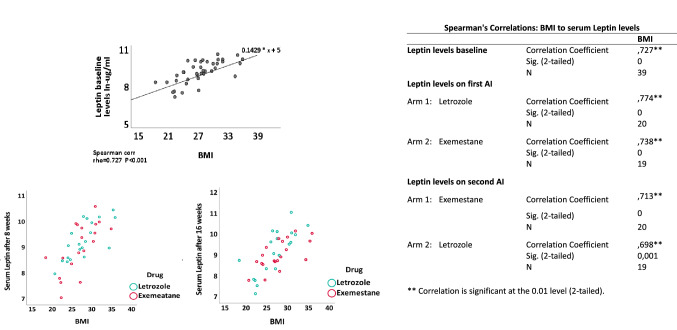
Correlation between plasma leptin levels (baseline) and body mass index (BMI). Dot plot to illustrate Spearman correlation between BMI (x-axis) and serum levels of leptin (y-axis) in breast cancer patients

Importantly, serum levels of leptin were found to be significantly decreased during treatment with exemestane compared to treatment with letrozole (*p* < 0.001), regardless whether exemestane was given as first or second therapy (Fig. 3). Our analysis also showed that treatment with letrozole slightly increased serum leptin levels without reaching the level of statistical significance. The leptin baseline levels showed a strong correlation to the body mass index (BMI) of the patients (rho = 0.7, *p* = 0.001) as expected. The correlations between BMI and leptin levels at baseline and over time are summarized in Fig. 4. All in all, BMI and plasma leptin levels were highly correlated throughout both treatment arms. Concerning the other adipokines, we also observed a trend towards a reduction of serum levels of adiponectin during treatment with exemestane, however, without reaching the level of statistical significance. No significant changes in serum levels of any other adipokines were registered during treatment with aromatase inhibitors.

**Fig. 5:**
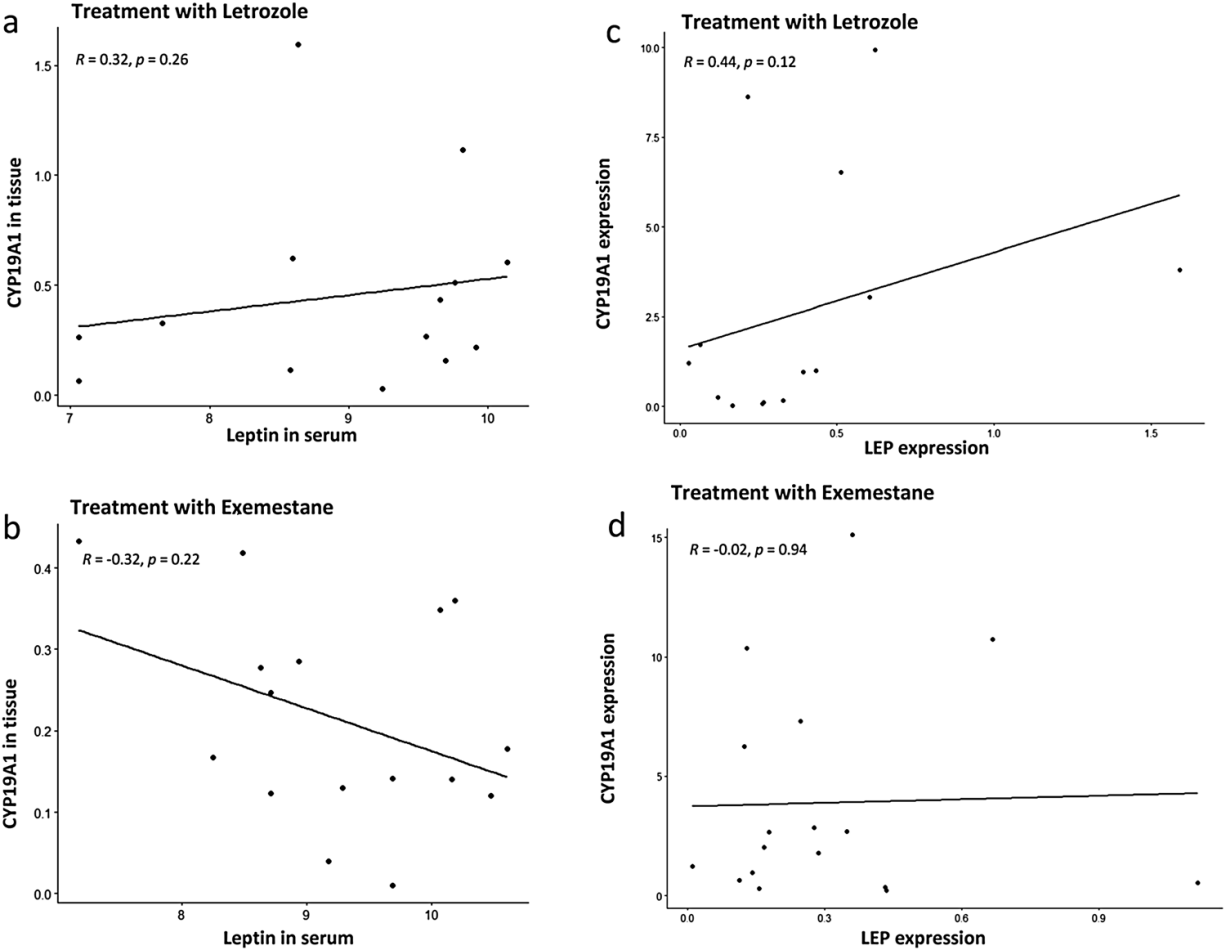
Correlation between CYP19 (aromatase) expression and leptin blood levels as well as leptin gene expression. The correlation between leptin levels in serum and CYP19 (aromatase) expression in tumor tissue is given during therapy with letrozole (**a**) and exemestane (**b**). In addition, the correlation between the leptin gene (LEP) expression in tumor tissue and CYP19 (aromatase) expression in the same tumor specimens in given while on treatment with letrozole (**c**) and exemestane (**d**)

To further investigate the relationship between leptin serum levels, leptin gene expression in tumor tissue and CYP19 (aromatase) expression in corresponding tumor specimens, we calculated the correlations between these parameters using whole genome sequencing data that were available for these patients. The findings are summarized in Fig. 5. Briefly, our results suggest a strong suppression of CYP19 expression in the tumor tissue during treatment with exemestane even in women with elevated leptin levels (Fig. 5b). In contrast, increasing leptin serum concentrations were found to associated with increasing CYP19 expression during therapy with letrozole (Fig. 5a).

In addition to the already mentioned disturbances in the adipokine levels, we observed that some cytokines belonging to the TNF superfamily were found to be significantly (*p* < 0.01) decreased during letrozole therapy while increased during exemestane treatment. These included TNF alpha, TNF Receptor Superfamily Member 8 (TNFRSF8) or sCD30, and TNF Superfamily Member 13B (TNFS13B/BAFF). The findings are summarized in Supplementary Fig. 2. Among all studied members of the IL-10 family of cytokines, we found only IL-11 to be significantly increased during letrozole therapy.

Among the other “regular” cytokines, we found IFN-beta, IL-12(p40) and VEGF to be significantly influenced by the aromatase inhibitors used in our trial (Table 2). Treatment with either letrozole or exemestane did not cause significant changes in all other cytokines measured in this study (sCD163, Chitinase 3-like 1, gp130, IFN-alpha2, IFN-gamma, IL-2, sIL-6Ralpha, IL12(p70), IL32, IL34, IL35, IP10, PDGF-bb, osteocalcin, osteopontin, pentraxin-3, TSLP, IL-24).

**Fig. 6:**
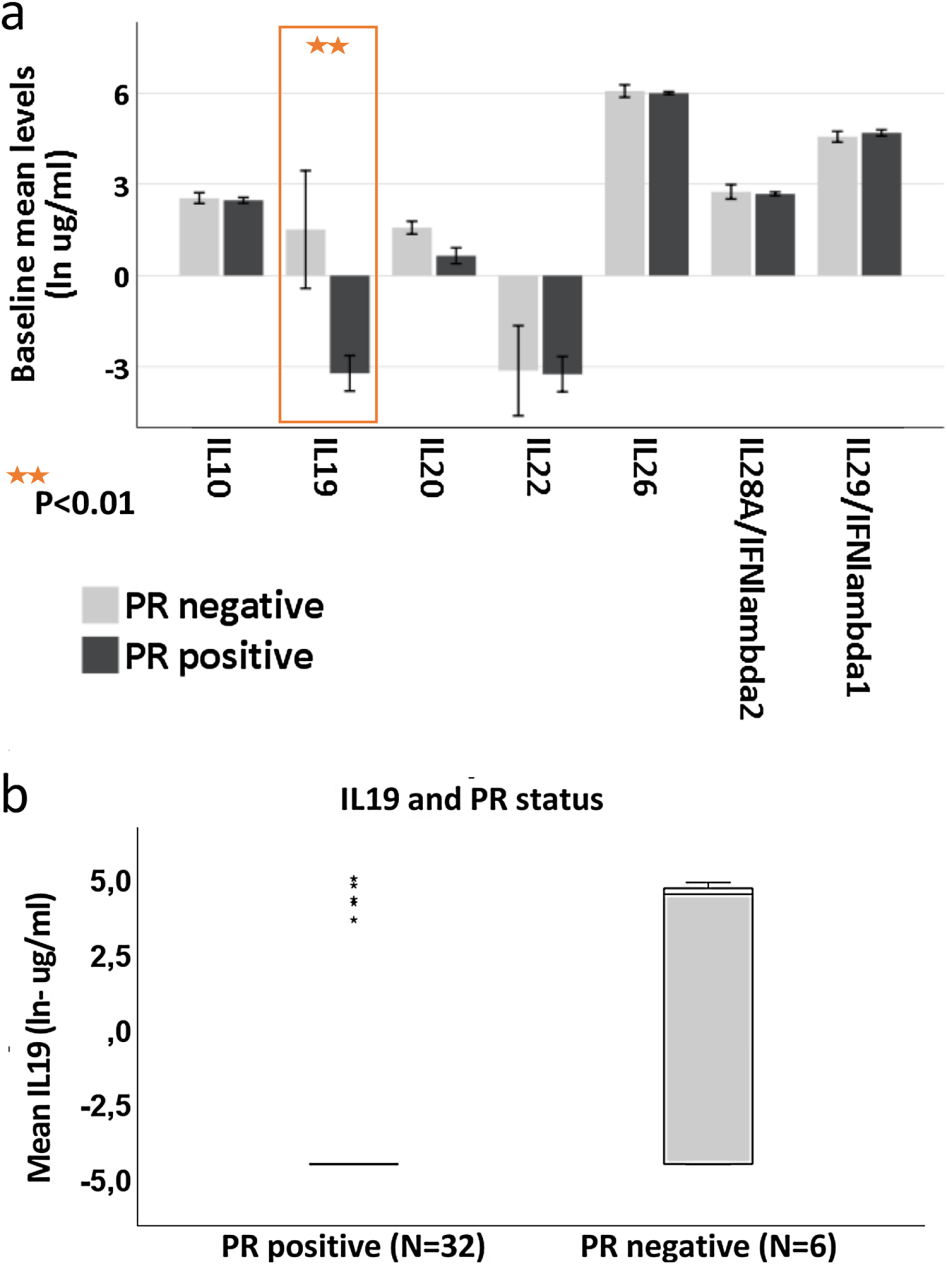
Correlation between PR expression in breast cancer tissue specimens and selected cytokine levels. The serum levels of IL19 were found to be significantly higher (*p* < 0.01) in the samples obtained from patients harboring a progesterone receptor (PR) negative tumor (*n* = 6) compared to patients with PR positive tumors (*n* = 32)

The progesterone receptor (PR) status varied among patients participating in this study as expected for this subgroup of patients (Table 1). Interestingly, one member of the IL-10 cytokine family, interleukin 19 (IL-19), showed significant variations in serum levels relative to PR status. Thus, patients with PR negative breast cancer had significantly higher levels of IL-19 compared to patients with PR positive breast cancer (*p* < 0.01). The findings concerning relations between the PR tumor status and cytokine levels are summarized in Fig. 6.

Finally, we were also able to investigate the influence of letrozole and exemestane on MMPs and found significant differences (*p* < 0.01) comparing the levels of MMP1 and MMP3 while on treatment with the two AIs (summarized in: Table 2).

## Discussion

While aromatase inhibitors are widely established as standard of care in all phases of ER positive breast cancer variants in postmenopausal women, several important questions are still unanswered concerning their basic mechanisms of action in vivo. Thus, a lack of cross-resistance between non-steroidal aromatase inhibitors (like letrozole) and steroidal aromatase inactivators (like exemestane) has been well-documented during treatment of metastatic breast cancer [10, 12–16, 19, 32]. This phenomenon has caused the establishment of exemestane therapy, either as monotherapy or in concert with mTOR-inhibitors like everolimus, following progression on non-steroidal AIs (letrozole/anastrozole) in the recommended treatment guidelines in many countries worldwide. To explore the potential mechanisms behind this important clinical observation, probably based on a fundamental, although not understood difference between the two major classes of AIs, we conducted the NEOLETEXE-trial at our institution. The study allows a direct head-to-head comparison of two of the most widely used aromatase disrupting agents, letrozole and exemestane. The results of a pre-planned substudy focusing on the influence of AI therapy on cytokines in general and adipokines in particular are given here.

Most interestingly, we present here evidence for a strong negative effect of exemestane therapy on serum leptin levels that is strikingly contrasted by the absence of this effect during letrozole therapy when given to the same patients in sequence (Fig. 3). This finding was not at all depending on the sequence of drugs as it could be shown in both treatment arms independent whether exemestane was given as the first of second treatment in the neoadjuvant setting. While the regulation of leptin levels by steroids is still a controversial point [33], androgens have been suggested to decrease plasma leptin levels, while estrogens are believed to increase leptin levels in vivo [34–38]. Thus, one possible explanation for the observed decrease of plasma leptin levels during monotherapy with exemestane seen in our study may be due to the androgenic effects of exemestane and its major metabolite 17-hydroxy-exemestane [39]. Estrogen suppression alone did not decrease leptin levels in vivo as documented by a modest increase of leptin serum concentrations during monotherapy with the extremely potent aromatase inhibitor letrozole in our study. In contrast to the steroidal compound exemestane, letrozole belongs to the pharmacological group of triazoles and does not exert androgen effects in vivo.

Our results indicate in fact a dual mode of action of exemestane in vivo. First, exemestane is working as a classical steroidal aromatase inactivator, causing metabolism of an aromatase molecule following binding of exemestane [4]. This reaction is also called “suicide-binding” in the literature [40]. In addition, our results presented here suggest an additional effect on aromatase expression due to the negative effect on leptin levels. Leptin has been shown to enhance aromatase expression via AP-1 in the MCF-7 cell line [27]. As a consequence, exemestane, in addition to be a classical aromatase inactivator, probably downregulates aromatase synthesis directly by suppression of leptin effects on promoter II and I.3 of the CYP19 gene. This additional effect of exemestane on the CYP19 promoter level is not present during therapy with letrozole, according to our findings presented here and may, at least partly, explain why exemestane may cause clinical responses in breast cancer patients who experience disease progression while on treatment with a non-steroidal aromatase inhibitor. We also investigated the correlation between leptin serum levels and leptin gene expression and CYP19 (aromatase) gene expression in corresponding tumor tissue specimens using whole genome sequencing (WGS) data. Briefly, our findings suggest a strong suppression of CYP19 gene expression in the tumor tissue during treatment with exemestane even in women with elevated leptin levels (Fig. 5b). In contrast, increasing leptin serum levels were found to be associated with increasing CYP19 expression during letrozole therapy.

Moreover, it has been shown by Catalano et al. [41] that leptin is able to induce a functional activation of ER alpha in MCF-7 cells via ERK1/ERK2 signaling. Thus, additional beneficial effects of leptin suppression by exemestane, not related to CYP19 regulation, cannot be entirely ruled out and may contribute to the reported clinical effects of exemestane following non-steroidal AIs.

Treatment with exemestane also caused a trend towards a (non-significant) suppression of serum adiponectin not observed during letrozole therapy. Adiponectin is produced by metabolically active white adipose tissue and inversely associated with adiposity. Adiponectin is believed to reduce the risk and the progression of breast cancer via its antiproliferative and possibly pro-apoptotic effects on breast cancer cells [42, 43]. No other adipokines were considerably affected by treatment of exemestane or letrozole in this trial.

Our cytokine panels allowed us also to investigate the potential effects of letrozole and exemestane on selected members of the Tumor Necrosis Factor (TNF) superfamily. While all members of the TNF superfamily, consisting of 19 ligands and 29 receptors [44], have been suggested to be involved in a variety of cellular events including proliferation, differentiation and apoptosis, the B-cell activating factor of TNF family (BAFF) has recently gained extra attention due to its major role in regulating the tumor microenvironment including induction of apoptosis [45, 46]. In the present study we found sCD30/TNFRSF8 to be significantly increased during exemestane therapy (*p* < 0.003) while it was not modified by letrozole. Thus, our findings indicate distinct influences of different aromatase inhibitors on key regulators of inflammation and immunity in human breast cancer that deserve further investigation.

The patients enrolled in the NEOLETEXE-trial were typical luminal-A breast cancer patients with highly ER positive breast tumors with co-expression of PR in 31 of 39 individual cases. However, a sub-population with ER positive/PR negative tumors in our trial (*n* = 8) gave us the opportunity to study cytokine profiles in these two subgroups of patients. Although the patient numbers are actually small, we found significantly elevated IL-19 levels in patients with ER positive / PR negative BC when compared to ER pos./PR positive cases (*p* < 0.01). Interleukin 19 is a cytokine belonging to the IL-10 family with multiple roles in immune regulation [47, 48]. In breast cancer, interleukin 19 seems to play an especially important role in disease progression [49]. Hsing et al. recently showed that upregulated IL-19 is associated with poor clinical outcome in BC patients [50]. It is well-known that loss of PR is indicating a worse prognosis in BC patients compared to PR positive cases, in general. The precise link between PR negativity and elevated IL-19 levels is currently unknown and will be investigated in follow-up studies.

Finally, we studied the effects of neoadjuvant letrozole and exemestane on metalloproteinases (MMPs). Breast cancer cells may release metalloproteinases to degrade matrix macromolecules, allowing the invasion of tissue barriers, blood vessels and lymph channel walls [51–54]. In our study, we were able to study the effects of AI therapy on three pivotal metalloproteinases simultaneously: MMP1 (collagenase-1), MMP2 (gelatinase-A) and MMP3 (stromelysin-1). We found a significant suppression of MMP1 during exemestane therapy when compared to letrozole (*p* = 0.023) and a significant suppression of MMP3 during therapy with letrozole (*p* = 0.003) when compared to exemestane, while the findings for MMP2 were not significantly different between the two AIs. Our findings are contrasting findings made by others, however obtained in vitro, showing that letrozole may decrease MMP2 levels [55]. Thus, aromatase inhibitors may have additional positive treatment effects on metalloproteinases by decreasing their levels in vivo as indicated here. These findings have to be confirmed in larger cohorts in the future.

In conclusion, treatment with exemestane significantly lowered serum leptin levels in breast cancer patients while letrozole did not. Our findings suggest a dual mode of action for exemestane, downregulating CYP-19 (aromatase) expression in the presence of high leptin levels probably due to effects on the CYP19 gene promoter involvement in addition to the well-known effects as a classical aromatase inactivator. The important role of leptin in breast cancer carcinogenesis and progression may influence the choice of aromatase inhibitors based on their distinct influence on adipokines like leptin in vivo. All in all, our results suggest a potential role for exemestane, especially in obese postmenopausal women who are typically harboring elevated leptin levels.

## Supplementary Information

Below is the link to the electronic supplementary material.

Supplementary file1 (PDF 3509 kb) **Suppl. Figure 1 **Spearman correlations between serum levels of adipokines during treatment with letrozole and exemestane. Spearman correlations between serum levels of 12 adipokines (*n* = 39) during therapy with letrozole and exemestane are shown by correlation dot plots, as well as by correlation coefficient values (rho) with significance (*p*) values. Significant *p* values are highlighted bold

Supplementary file2 (PDF 54 kb) **Suppl. Figure 2 **Influence of treatment with letrozole and exemestane on members of the TNF-cytokine-family. Serum levels of three members of the TNF-cytokine family (BAFF/TNFSF13B = B cell activating factor of the TNF-family; sCD30 = soluble CD30 or TNFRSF8 = Tumor necrosis Factor Receptor Superfamily, member 8 and TNF-alpha) were significantly higher (*p* < 0.01 for all) while on exemestane therapy compared to letrozole therapy.

## Data Availability

Any requests for additional data or supporting material will be considered for qualified external researchers who provide a methodologically sound proposal. Proposals should be directed to the corresponding author in the first instance.

## Abbreviations

AIs: Aromatase inhibitors
AP-1: Activator protein 1
CYP19: Cytochrome P-450 19 (aromatase)
ER: Estrogen receptor
FSH: Follicle-stimulating hormone
HER-2: Human epidermal growth factor receptor
IGF-I: Insulin-like-growth factor I
INF: Interferon
LH: Luteinizing hormone
IRS: Insulin receptor substrate
JAK: Janus tyrosine kinase
LABC: Locally advanced breast cancer
MAPK: Mitogen-activated protein kinase
MMPs: Matrix metalloproteinases
MRI: Magnetic resonance imaging
NET: Neoadjuvant endocrine therapy
PDGF: Platelet-derived growth factor
PR: Progesterone receptor
SOCS: Suppressor of cytokine signaling
STAT: Signal transducer and activator of transcription
TNF: Tumor necrosis factor
VEGF: Vascular endothelial growth factor
