# Supplementary material for: Lack of cross-resistance between non-steroidal and steroidal aromatase inhibitors in breast cancer patients: the potential role of the adipokine leptin
Source: Breast Cancer Res Treat. 2021 Sep 23;190(3):435–49. doi: 10.1007/s10549-021-06399-x (PMC8558290; doi:10.1007/s10549-021-06399-x)
Supplement: Supplementary file 1 — Supplementary file1 (PDF 3509 kb) Suppl. Figure 1 Spearman correlations between serum levels of adipokines during treatment with letrozole and exemestane. Spearman correlations between serum levels of 12 adipokines (n = 39) during therapy with letrozole and exemestane are shown by correlation dot plots, as well as by correlation coefficient values (rho) with significance (p) values. Significant p values are highlighted bold [file 10549_2021_6399_MOESM1_ESM.pdf]

Suppl. Fig. 1

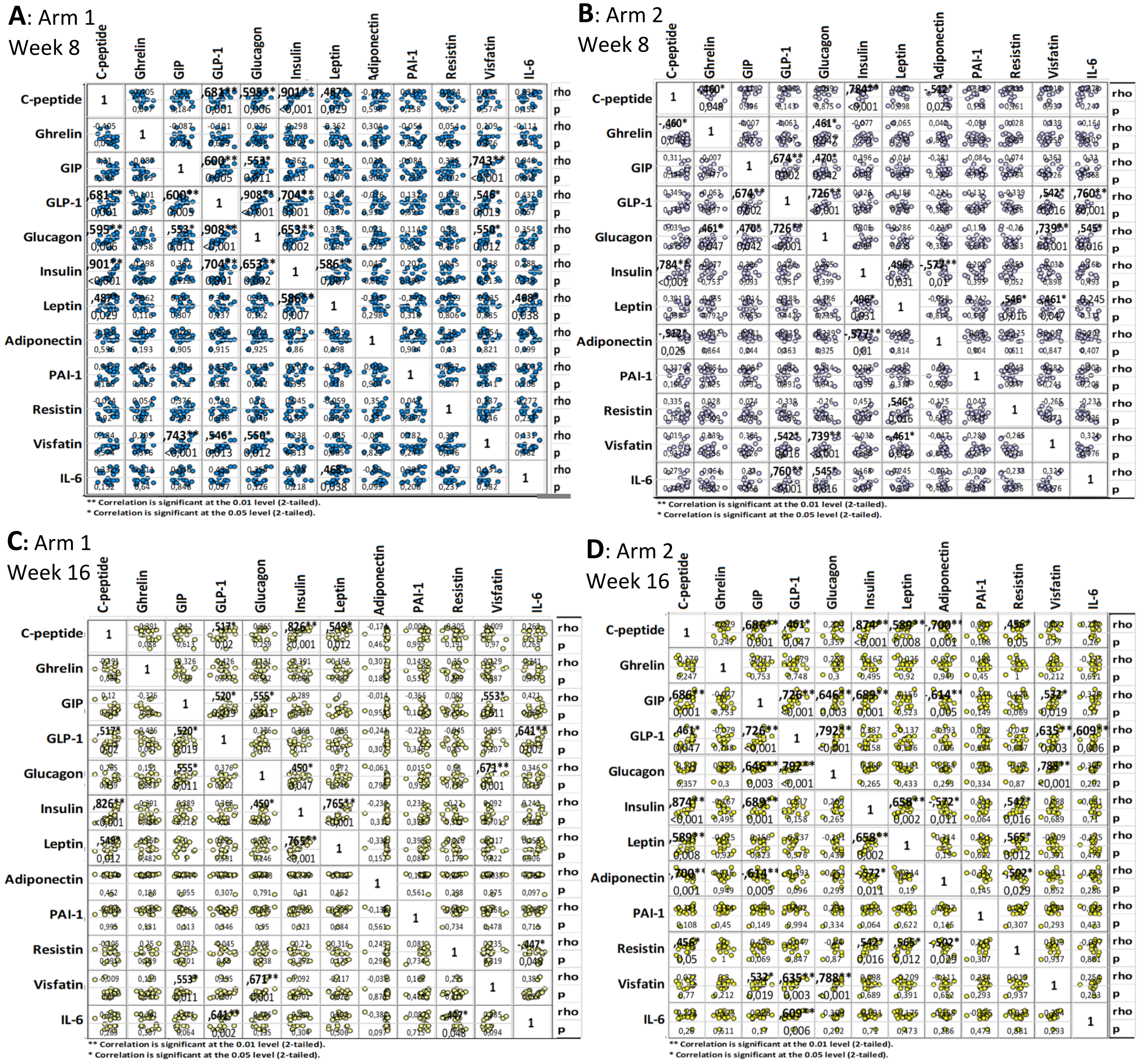

Treatment arm 1 = Letrozole for 8 weeks followed by Exemestane for another 8 weeks

Treatment arm 2 = Exemestane for 8 weeks followed by Letrozole another 8 weeks
