# Supplementary figures and images for: Lack of cross-resistance between non-steroidal and steroidal aromatase inhibitors in breast cancer patients: the potential role of the adipokine leptin
Source: Breast Cancer Res Treat. 2021 Sep 23;190(3):435–49. doi: 10.1007/s10549-021-06399-x (PMC8558290; doi:10.1007/s10549-021-06399-x)

Suppl. Fig. 2

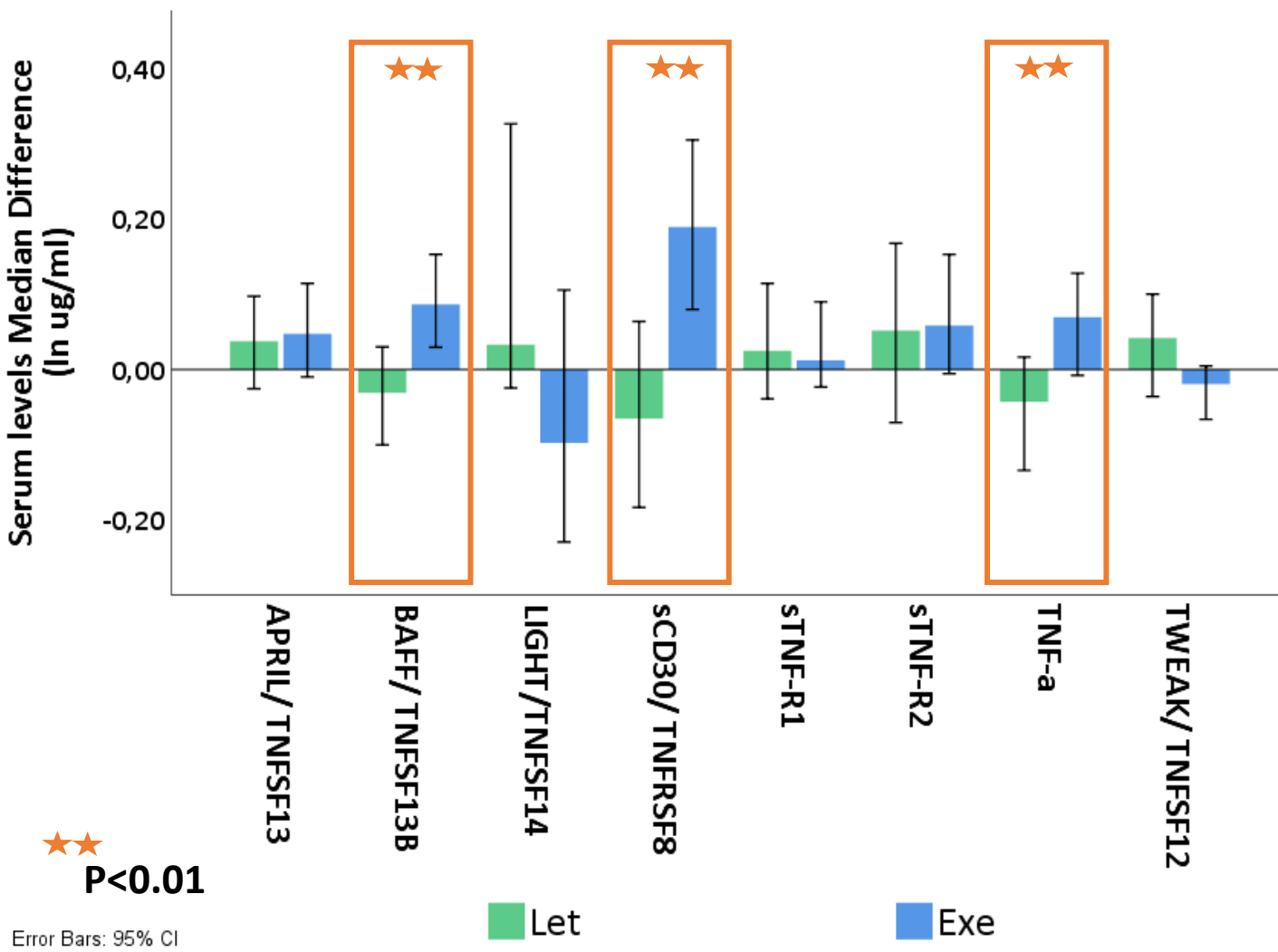

Supplement: Supplementary file 2 — Supplementary file2 (PDF 54 kb) Suppl. Figure 2 Influence of treatment with letrozole and exemestane on members of the TNF-cytokine-family. Serum levels of three members of the TNF-cytokine family (BAFF/TNFSF13B = B cell activating factor of the TNF-family; sCD30 = soluble CD30 or TNFRSF8 = Tumor necrosis Factor Receptor Superfamily, member 8 and TNF-alpha) were significantly higher (p < 0.01 for all) while on exemestane therapy compared to letrozole therapy. [file 10549_2021_6399_MOESM2_ESM.pdf]
